# The *rph2* Gene Is Responsible for High Level Resistance to Phosphine in Independent Field Strains of *Rhyzopertha dominica*

**DOI:** 10.1371/journal.pone.0034027

**Published:** 2012-03-26

**Authors:** Yosep S. Mau, Patrick J. Collins, Gregory J. Daglish, Manoj K. Nayak, Paul R. Ebert

**Affiliations:** 1 School of Integrative Biology, The University of Queensland, Saint Lucia, Queensland, Australia; 2 Faculty of Agriculture, The University of Nusa Cendana, Kupang, Nusa Tenggara Timur, Indonesia; 3 Department of Employment, Economic Development and Innovation, Ecosciences Precinct, Brisbane, Queensland, Australia; 4 Cooperative Research Centre for National Plant Biosecurity, Bruce, Australian Capital Territory, Australia; Kyushu Institute of Technology, Japan

## Abstract

The lesser grain borer *Rhyzopertha dominica* (F.) is one of the most destructive insect pests of stored grain. This pest has been controlled successfully by fumigation with phosphine for the last several decades, though strong resistance to phosphine in many countries has raised concern about the long term usefulness of this control method. Previous genetic analysis of strongly resistant (SR) *R. dominica* from three widely geographically dispersed regions of Australia, Queensland (SR_QLD_), New South Wales (SR_NSW_) and South Australia (SR_SA_), revealed a resistance allele in the *rph1* gene in all three strains. The present study confirms that the *rph1* gene contributes to resistance in a fourth strongly resistant strain, SR2_QLD_, also from Queensland. The previously described *rph2* gene, which interacts synergistically with *rph1* gene, confers strong resistance on SR_QLD_ and SR_NSW_. We now provide strong circumstantial evidence that weak alleles of *rph2*, together with *rph1*, contribute to the strong resistance phenotypes of SR_SA_ and SR2_QLD_. To test the notion that *rph1* and *rph2* are solely responsible for the strong resistance phenotype of all resistant *R. dominica*, we created a strain derived by hybridising the four strongly resistant lines. Following repeated selection for survival at extreme rates of phosphine exposure, we found only slightly enhanced resistance. This suggests that a single sequence of genetic changes was responsible for the development of resistance in these insects.

## Introduction

Phosphine (PH_3_) is the most economically viable fumigant for the control of insect pests of stored grain, making it the major method of control worldwide [1], [2], [3], [4], [5]. Low level resistance to phosphine was first reported in an FAO global survey undertaken in the 1970s [6]. Widespread high level resistance to phosphine in recent years now threatens the continued use of this chemical [7]. The lesser grain borer *Rhyzopertha dominica* (F.) is one of the most destructive pests of stored grains and high levels of phosphine resistance have been reported from several countries, such as Bangladesh [8], Brazil [9], [10], India [11], [12], China [13], [14], and the Philippines [15]. In Australia, strongly resistant *R. dominica* was first detected in southern Queensland in 1997 [16], followed by detection of another strongly resistant strain 300 km to the north in 1998 (Collins, unpublished). Emergence of strongly phosphine resistant strains of *R. dominica* was recently reported in New South Wales [17] and in South Australia (Wallbank, personal communication).

Genetic analysis of the initial strongly resistant strain from southern Queensland SR_QLD_, (elsewhere referred to as QRD569), identified two major genes responsible for resistance [3], [18]. Molecular genetic analysis demonstrated that the strong resistance of SR_QLD_ was provided by synergistic interaction between the two genes, *rph1* and *rph2* [19]. Schlipalius et al. [19] also showed that *rph1* is the major gene responsible for the weak resistance phenotype of the strain WR_QLD_, previously referred to as QRD369 [3].

Comparative genetic analysis [20] revealed that two major genes are responsible for the strong resistance phenotype of insects from more recent resistance outbreaks in New South Wales (SR_NSW_) and South Australia (SR_SA_). Results of complementation analysis involving the weakly resistant strain WR_QLD_ suggest that the gene responsible for the weak resistance phenotype, *rph1*, contributes to resistance in both SR_NSW_ and SR_SA_. Interaction between *rph1* and a second gene(s) is likely responsible for the strong resistance phenotype exhibited by these strains. However, it was not determined whether or not any genes, other than *rph1*, were shared between the two strains. To date, the genetics of resistance in the second strong resistance strain collected from southern Queensland (referred to as SR2_QLD_ in the remainder of this report) has not been determined. Thus, it was not known whether the *rph1* and *rph2* genes originally identified in SR_QLD_ also were responsible for resistance in SR2_QLD_.

This paper reports the genetic analysis of SR2_QLD_ as well as three additional strains that had been found previously to contain a resistance allele at the *rph1* locus. These four strongly resistant strains, SR_QLD_, SR2_QLD_, SR_SA_, and SR_NSW_, originate from four widely separated geographical regions of Australia. They have not previously been analysed to determine whether the *rph2* gene originally identified in SR_QLD_ also contributes to their resistance phenotype. Knowing the genetic basis of resistance in these strains will allow us to predict how resistance will develop in the field. We also have created a laboratory strain that contains the resistance alleles from each of the four strongly resistant strains. Our results are consistent with the same two genes contributing to resistance in each of the four resistance outbreaks. Not surprisingly, crossing these strains and reselecting for high level resistance does not result in a major increase in the resistance level.

## Materials and Methods

### Insect Strains

Four strongly resistant strains (SR_NSW_, SR_SA_, SR_QLD_ and SR2_QLD_) and a weakly resistant strain (WR_QLD_) were employed in this study. Two strains (SR_NSW_, SR_SA_) were collected from Merriwagga in south-western New South Wales (1999) and Port Adelaide in South Australia (2000), respectively, and were assigned the following collection reference numbers: NNRD2864 and NSRD3075 [17]. Strains SR_QLD_ and SR2_QLD_ were collected from Millmerran (1997) and Wandoan (1998) in southern Queensland and were assigned collection reference numbers QRD569 and QRD676, respectively. The weakly resistant strain (WR_QLD_) was also collected from Millmerran (1997) and was assigned reference number QRD369. The approximate distance between the geographic origins of any two strongly resistant strains was 250–1500 km. All resistant strains were selected with phosphine for at least five generations to promote homozygosity at resistance loci. All strains were cultured on whole wheat and maintained at 30°C and 55% relative humidity.

### Complementation and allelic relationship analysis

Complementation analysis was carried out to determine whether the *rph1* gene that controls weak resistance to phosphine in WR_QLD_ and contributes to strong resistance in SR_QLD_ [19], also contributes to strong resistance in the second strongly resistant strain from Queensland, SR2_QLD_. The test involved crossing WR_QLD_ and SR2_QLD_ and determining the resistance phenotype of the F_1_ and F_2_ progeny as previously described for SR_QLD_ [3]. In addition, crosses between all four strongly resistant strains were made to determine the relationships between their respective resistance alleles. Crosses representing all six pairwise strain combinations were produced from the four strongly resistant strains: SR_NSW_xSR_SA_, SR_QLD_xSR_NSW_, SR_QLD_xSR_SA_, SR_QLD_xSR2_QLD_, SR_NSW_xSR2_QLD_ and SR2_QLD_xSR_SA_. F_1_ and F_2_ progenies were generated from each cross and the response to phosphine exposure of individuals within these progenies were examined to determine the relationships between the resistance genes of the parental strains.

### Interaction between resistance genes when combined in a single strain

We also determined whether a strain could be selected with enhanced resistance to phosphine from a population containing all resistance genes from each of the four independently derived strongly resistant strains employed in this study. To combine all the resistance genes from the four strains in a single population, we employed two different crossing strategies; combined crosses and double crosses.

### Combined crosses

Combined crosses provided the easiest way to introduce all resistance genes into a single population. Initially, two pairwise crosses were made from the four parental strains, SR_QLD_xSR_SA_ and SR_NSW_xSR2_QLD_, by mating an adult virgin female with an adult male on kibbled grain inside a plastic capsule. Five identical matings were set up for each of the two crosses. After two weeks, the adults were removed to fresh kibbled grain and the old grain containing the progeny from each set of five identical matings was pooled in a single plastic cup. This cup was topped up with fresh kibbled grain and the F_1_ progeny were allowed to mature. Fifty mature (1–2 weeks post eclosion) F_1_ progeny from each of the two crosses were combined and allowed to mate freely for two weeks to produce the combined progeny. This is referred to as the combined crosses (CC). Collectively, the progeny of the combined crosses would carry all resistance alleles from the four parental strains. The resulting F_1_ progeny of the combined crosses were, again, allowed to mate freely for two weeks to produce the F_2_ generation. Approximately 600 adult F_2_ progeny were allowed to mate freely to produce an F_3_ generation. Selections for phosphine resistance commenced in the F_3_ generation at a concentration of 0.5 mg/L phosphine. The survivors of this selection were retained to produce subsequent generations. The second and third selections were performed on the F_5_ and F_7_ generations, both at 1.0 mg/L phosphine. A parallel population of F_7_ individuals that had previously been subjected to two rounds of selection were also tested for their resistance phenotype. A second mortality response curve was also performed in the F_9_ generation, by which time the line had already been subjected to three rounds of phosphine selection.

### Double Hybrid Crosses

The second approach was to set up defined crosses in each of two generations to establish a doubly hybrid strain. Initially, two single crosses were made, SR_QLD_×SR_NSW_ and SR2_QLD_×SR_SA_. Subsequently, F_1_ individuals of the two single crosses were mated to produce double cross progeny. This ensured that the parental strains contributed equally to the genotypes present in the resulting progeny. These crosses involved the same four parental strains used for the combined crosses (CC) but in different pairwise combinations in case this influenced the likelihood of selecting specific resistance genotypes.

The initial crosses were carried out as reciprocal single pair crosses between virgin individuals, for a total of ten crosses for each pair of strains. The parents from the initial crosses were pooled on fresh kibbled grain to establish the two single cross strains (SR_QLD_×SR_NSW_) and (SR2_QLD_×SR_SA_). Reciprocal crosses were then set up between progeny of the initial crosses to create 20 double crosses, each of which produced progeny derived from all four parental strains. The adults were removed and the grain containing the eggs of 20 double cross mating pairs was combined in a bottle containing ∼200 g whole grain. The resulting adult (1–3 week post eclosion) F_1_ double cross progeny were transferred to new grain and left to mass cross for two weeks to produce an F_2_ generation. The F_3_ generation was produced through mass crosses of approximately 600 F_2_ adults. Successive generations were produced by mass crossing progeny of the previous generation. Selection for phosphine resistance was carried out on adults of the F_3_, F_5_ and F_7_ generations. Progeny of both the initial single crosses and subsequent double crosses were selected at 0.5 mg/L phosphine in the F_3_ generation, but 1.0 mg/L phosphine in the F_5_ and F_7_ generations. Phosphine resistance was quantified in the F_7_ and F_9_ generations.

### Phosphine fumigation

Phosphine resistance of the parental strains and the progeny of the crosses was measured by exposing insects to phosphine fumigation for 48 hours [3] at a range of phosphine concentrations (0.01–2.0 mg/L). Phosphine was generated in a collection tube containing aluminium phosphide introduced into a 5% sulphuric acid solution [21]. Phosphine concentration was determined by gas chromatography [Varian (Mount Waverley, Victoria, Australia) aerograph model 90-P] utilising dichlorofluoromethane (Refrigerant F24; Lovelock Luke, Mayne, Queensland, Australia) as the carrier gas and a gas density balance detector.

Adult beetles (1–3 weeks post eclosion) were confined within small plastic cups (50 beetles per cup) containing 5 g whole grain. The cups were placed inside gas-tight desiccators and phosphine was injected into the desiccators through a septum. The insects were exposed to phosphine for 48 hours at 25°C and 70% r.h. then held for 14 days at 25°C and 55% r.h. when end-point mortality was assessed. A minimum of 100 insects was fumigated at each phosphine concentration.

### Data analysis

Mortality data for each strain or hybrid line were subjected to log-concentration/probit-regression analysis [22]. Mortality data were first corrected for control mortality (≤10%) based on Abbott's formula [23]. The probit analysis was carried out using the GenStat7 statistical package [24]. The goodness-of-fit to the log-dose/probit mortality line was determined by a chi-square test. In the goodness-of-fit calculation, at doses where the expected response was less than one, the number of observed responses was combined with the value for an adjacent dose and the degrees of freedom for the chi-square analysis were adjusted accordingly. The resistance factors for both single cross and double cross progenies were calculated by dividing the LC_50_ of each progeny generation by the mean LC_50_ of the parental strains.

## Results

### Complementation and allelic relationship analysis

The resistance phenotype of the four strongly resistant *R. dominica* strains that have been characterised in Australia differ by at most three fold. The rank order of resistance is SR_SA_≅SR2_QLD_<SR_QLD_≅SR_NSW_. Previous work suggested that evolution of resistance was constrained in the order in which genetic changes could occur [19]; that two genes contributed to resistance in multiple instances and that at least one resistance gene was common to multiple highly resistant strains [20]. The following comparative genetic experiments use complementation tests and gene stacking to reveal previously unknown resistance mechanisms or previously unidentified resistance genes.

### The *rph1* gene contributes to resistance in SR2_QLD_

**Figure 1:**
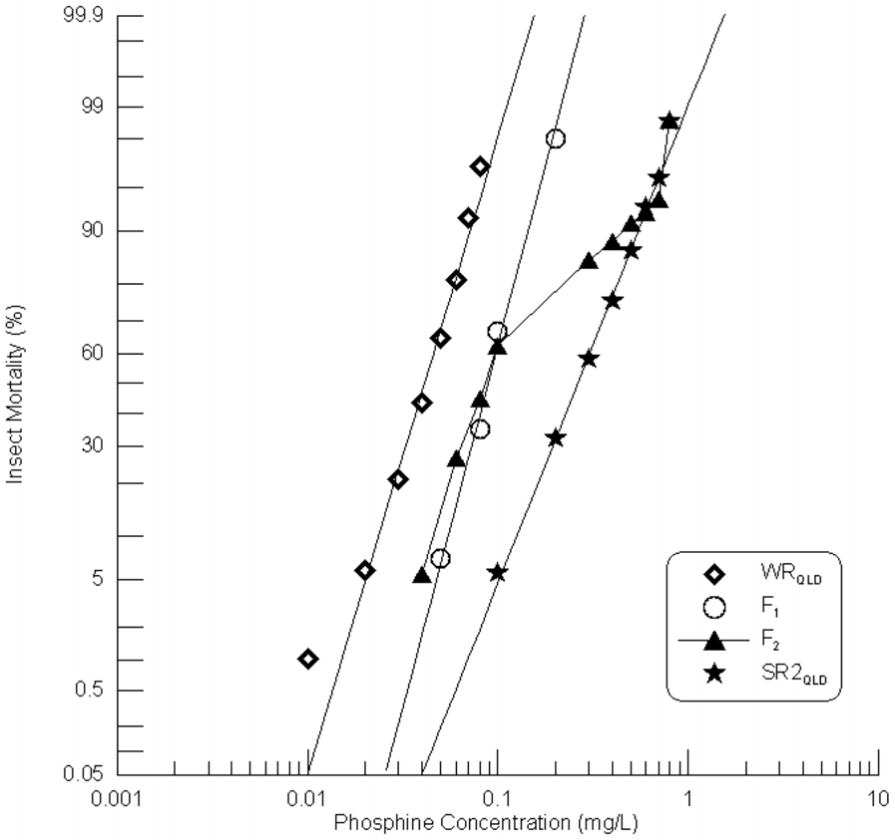
Resistance response of F_1_ hybrids and F_2_ progeny of a cross between a weakly resistant and a strongly resistant *R. dominica* strain from Queensland. Results are presented as log-dose mortality of the F_1_ hybrids and the F_2_ progeny with reference curves of the parental strains, WR_QLD_ (Weak R-Strain) and SR2_QLD_ (R-Strain). Phosphine exposure was for 48 hours at 25°C and 70% r.h.

The second strongly resistant strain from Queensland, SR2_QLD_, is distinct from SR_QLD_ that was analysed previously [18] (Schlipalius et al. 2002). SR2_QLD_ was crossed with the weakly resistant strain, WR_QLD_, which is homozygous for the resistance allele of the *rph1* gene. Probit analysis revealed that the responses of parental strains SR2_QLD_ and WR_QLD_ as well as their F_1_ progeny were linear (Figure 1). The relevant chi-square values of the response data were 1.407 (df = 7, p = 0.985) and 4.95 (df = 7, p = 0.666) for SR2_QLD_ and WR_QLD_ respectively, and 2.717 (df = 5, p = 0.744) for the F_1_.

The F_1_ progeny of this cross would be expected to exhibit a resistance phenotype at least as strong as the weakly resistant strain, WR_QLD_, if the *rph1* gene contributes to resistance in SR2_QLD_. If this gene does not contribute to resistance in SR2_QLD_, the hybrid progeny would be heterozygous for the incompletely recessive resistance alleles and nearly completely sensitive to phosphine. The F_1_ hybrids are more resistant than WR_QLD_, as expected if the *rph1* gene contributes to resistance in both parental strains (Figure 1).

### Genetic complementation analysis of *SR_NSW_*×*SR_SA_*

**Figure 2:**
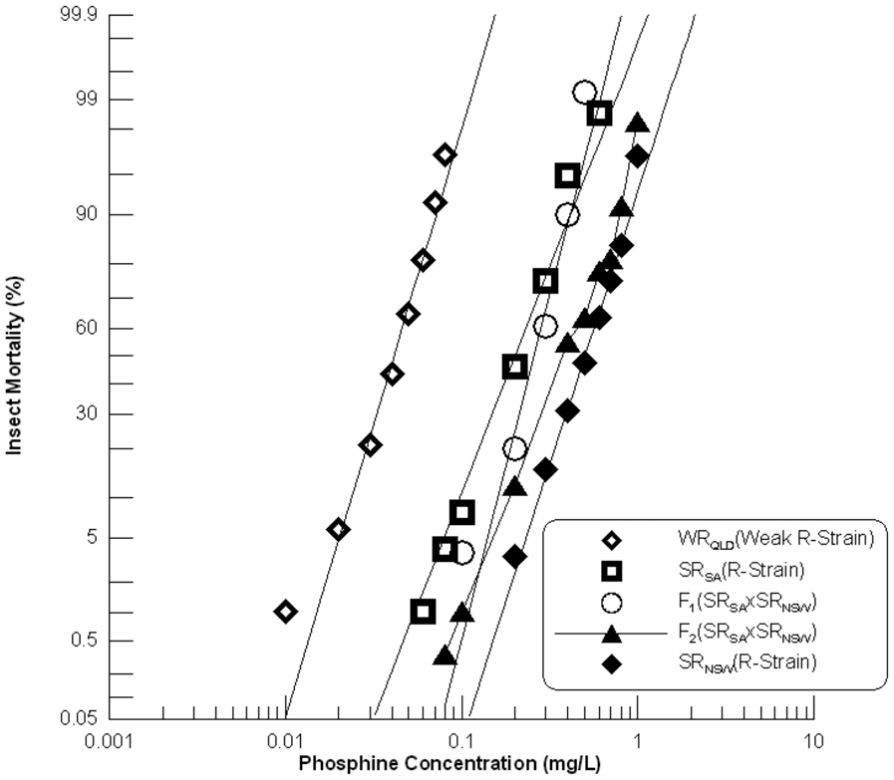
Resistance response of F_1_ hybrids and F_2_ progeny of a cross between strongly resistant *R. dominica* strains from South Australia and New South Wales. Results are presented as log-dose mortality of the F_1_ hybrids and the F_2_ progeny, together with reference curves of the parental strains, SR_SA_ and SR_NSW_, and the weakly resistant strain from Queensland (WR_QLD_). Phosphine exposure was for 48 hours at 25°C and 70% r.h.

A resistance allele of the *rph1* gene was previously found to contribute to the phosphine resistance phenotype of both SR_NSW_ and SR_SA_ via complementation analysis with the weakly resistant strain WR_QLD_ [20]. In the present study, SR_NSW_ and SR_SA_ were crossed and their hybrid progeny analysed to confirm lack of complementation at *rph1*, as well as to determine relationships between resistance alleles at other loci. Probit analysis of both parental strains and their F_1_ progeny revealed linear responses (Figure 2, Table 1), indicating that the strains are homogenous with respect to their resistance phenotypes.

**Table 1:** Probit analysis of the response to phosphine exposure of four strongly resistant *R. dominica* strains, SR_QLD_, SR2_QLD_ SR_NSW_, SR_SA_, as well as their combined cross progenies.

*Strain (Cross)*	*n*	*Slope ± SE*	*LC_50_ (95% FL) (mg/L)*	*LC_99.9_ (mg/L)*	*df*	*χ^2^*	*P*
SR_SA_	2168	4.43*±*0.10	0.208 (0.204–0.216)	1.035	7	3.454	0.840
SR_QLD_	2125	4.39*±*0.15	0.412 (0.398–0.426)	2.086	8	5.261	0.729
SR2_QLD_	1144	4.02*±*0.21	0.261 (0.253–0.269)	1.537	7	1.407	0.985
SR_NSW_	2565	4.94*±*0.21	0.499 (0.483–0.516)	2.106	7	8.239	0.312
F_7_ (CC)+	1631	5.00*±*0.21	0.691 (0.661–0.721)	2.871	8	4.998	0.758
F_9_ (CC)	1914	5.33*±*0.20	0.901 (0.871–0.931)	3.424	8	5.811	0.668

Estimated lethal concentrations, slopes and goodness-of-fit tests of probit lines of the parental strains, F_7_ and F_9_ progenies are presented. Insects were exposed to phosphine at generations F_3_, F_5_ and F_7_ for 48 hours at 25°C and 70% r.h.

*Significant (P<0.05); **significant (P<0.01); ***significant (P<0.001).

+ CC = Combined Crosses [mass crosses between the F_1_ progeny of the following crosses: (SR_QLD_×SR_SA_) and (SR2_QLD_×SR_NSW_)].

The previous report that both strains contain resistance alleles of *rph1* suggests that the hybrid progeny should at least show a level of resistance equivalent to that of the reference strain, WR_QLD_, which is homozygous resistant at *rph1*. The F_1_ hybrid progeny actually show a much higher level of resistance than seen for WR_QLD_ (Figure 2), a level of resistance equivalent to that of the strongly resistant parental strain SR_SA_. As both strains are known to carry a semi-dominant resistance allele at a second locus [20], the results could be explained as an additive semi-dominant effect of two distinct genes (in addition to homozygosity at *rph1*). However, the resistance phenotype due to the second gene in each strain differs by about two fold. Thus, the results could also be explained as two distinct alleles in the same gene that, in combination, provide a level of resistance dictated by the weaker of the two alleles.

Interpretation of the F_2_ results is complicated by the fact that there is only a two fold difference in the LC_50_ between the parental strains, SR_SA_ and SR_NSW_. The narrow distance between the parental mortality curves makes it difficult to identify a plateau in the F_2_ response curve as an indicator of monogenic control of resistance (beyond that caused by the *rph1* locus). Because the level of resistance due to the second gene differs between the strains three distinct F_2_ genotypes would result, even if the second resistance factor in each strain was actually an allele of the same gene. This increases the difficulty of distinguishing a plateau in the curve. One thing that is clear is that none of the F_2_ individuals approach the sensitivity of WR_QLD_ as would be expected of 6.25% of the individuals if the second resistance factors were not allelic. The most reasonable conclusion is that the strong resistance phenotype exhibited by these strains is controlled by *rph1* together with a second gene that is common to the two strains, though the strengths of the alleles of the second gene differ considerably.

### Genetic complementation analysis of *SR_NSW_*×*SR_QLD_*

**Figure 3:**
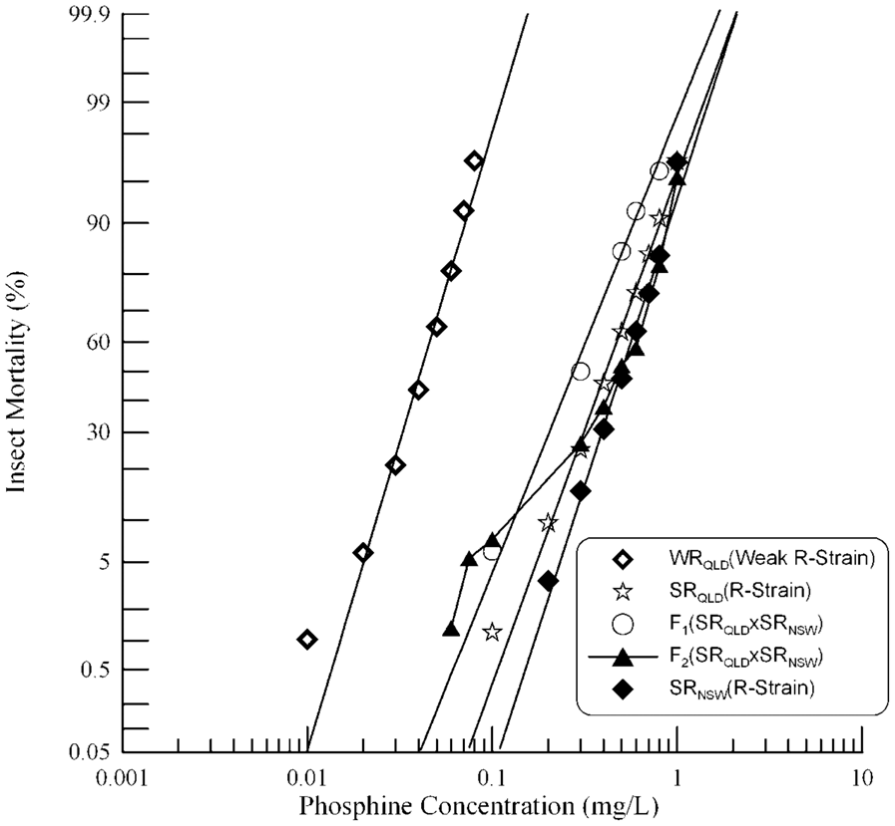
Resistance response of F_1_ hybrids and F_2_ progeny of a cross between strongly resistant *R. dominica* strains from Queensland and New South Wales. Results are presented as log-dose mortality of the F_1_ hybrids and the F_2_ progeny with reference curves of the parental strains, SR_QLD_ and SR_NSW_, and the weakly resistant strain from Queensland (WR_QLD_). Phosphine exposure was for 48 hours at 25°C and 70% r.h.

SR_NSW_ was also crossed to SR_QLD_, a well-characterised strongly resistant strain and the first strongly resistant strain collected in Australia. Responses of SR_NSW_ and SR_QLD_, as well as their F_1_ progeny were linear (Figure 3, Table 1). Response curves of the parental strains are almost overlapping, indicating a very similar level of resistance between the two strains, with SR_NSW_ only 1.2 times the resistance of SR_QLD_. The hybrid of this cross is slightly more sensitive to phosphine than is either parent, though it is still much more resistant (>10-fold) than the weakly resistant strain WR_QLD_. Both parents are known to be homozygous for a recessive resistance allele at *rph1* [3], [20] as well as homozygous for a second gene that is weakly semi-dominant. The semi-dominance can not explain the very high level of resistance in the F_1_ progeny rather, the resistance is equivalent to the synergistic action between the *rph1* and *rph2* genes previously reported for SR_QLD_ [19]. Thus, the strong resistance exhibited by the F_1_ progeny of this cross suggests that, as with SR_QLD_, *rph1* and *rph2* are responsible for resistance in SR_NSW_. The lack of a minor effect resistance factor that is unique to one or the other parental strain could completely explain the very minor decrease in resistance of the hybrid progeny relative to their parents.

As with the F_1_ progeny, a small proportion (5%–25%) of the F_2_ progeny are more sensitive than the parental strains. Most of the F_2_ progeny, on the other hand, show a resistance level between those of the parental strains (Figure 3). The F_2_ progeny would be expected to form a plateau at ∼75% mortality if a single gene in addition to *rph1*, controls resistance in the stronger resistant parent SR_NSW_. However, as the response curves of both parental strains are overlapping, it is impossible to determine whether a plateau occurs at this point. Interpretation of the F_2_ results is further complicated by the weak incomplete recessivity of *rph2* in SR_QLD_ [3], [18] and a second resistance gene (possibly *rph2*) in SR_NSW_ [20]. On balance, the F_2_ results are completely consistent with the F_1_ data, and indicate that a resistance allele of *rph2* does indeed contribute to resistance in SR_NSW_. The results also suggest that the previously noted minor, dominant resistance factor from one of the parents is likely the product of a single gene.

### Genetic complementation analysis of *SR_QLD_×SR_SA_*

**Figure 4:**
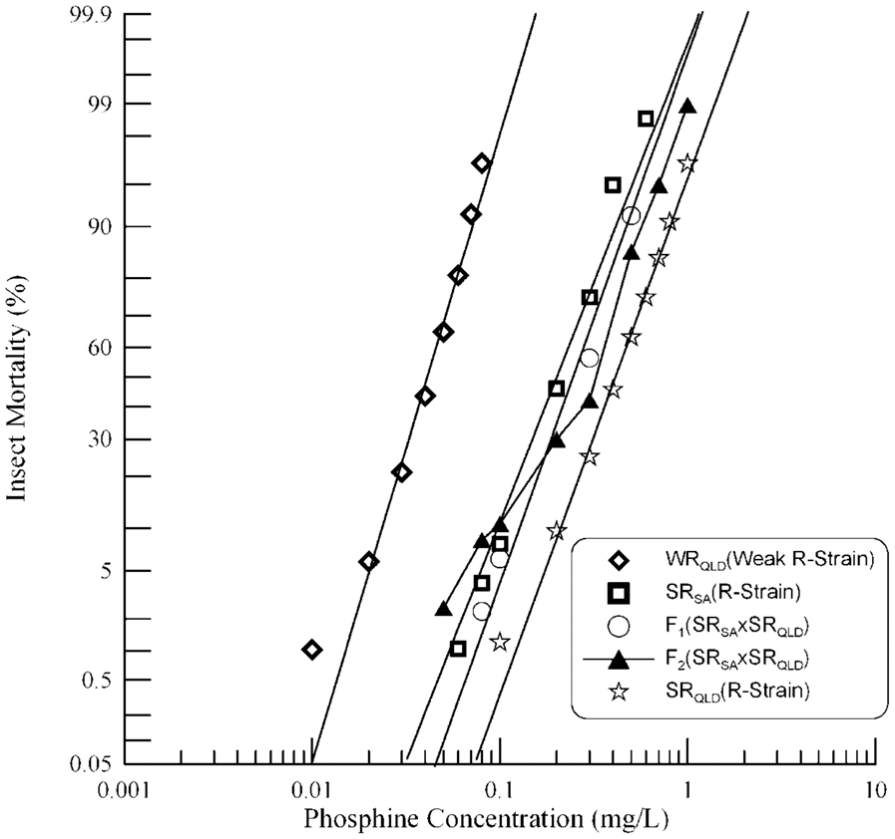
Resistance response of F_1_ hybrids and F_2_ progeny of a cross between strongly resistant *R. dominica* strains from South Australia and Queensland. Results are presented as log-dose mortality of the F_1_ hybrids and the F_2_ progeny with reference curves of the parental strains, SR_SA_ and SR_QLD_, and the weakly resistant strain from Queensland (WR_QLD_). Phosphine exposure was for 48 hours at 25°C and 70% r.h.

As was done previously with the strongly resistant strain from New South Wales, the strongly resistant strain from South Australia, SR_SA_, was also crossed with its Queensland counterpart, SR_QLD_, which is homozygous for both phosphine resistance genes *rph1* and *rph2*. Probit analysis of the response to phosphine of the parental strains and F_1_ progeny of this cross indicate a homogeneous response (Figure 4). Unlike the previous SR_QLD_×SR_NSW_ cross in which the parental strains were nearly equally resistant, the resistance phenotype of the SR_SA_ strain is less than half that of the strain from Queensland, SR_QLD_. The F_1_ progeny exhibited a slightly more resistant phenotype than the parental strain SR_SA_. The response of the F_1_ is as would be expected from non-complementation at the *rph1* locus, indicating that each of the two parental strains is homozygous for a resistance allele of the *rph1* gene. This is consistent with a previous detailed analysis in which the *rph1* gene was found to contribute to resistance in each of SR_SA_, SR_NSW_ [20] and SR_QLD_ [18]. The additional resistance is presumably due to additional factors from the parental strains. Because of the comparative weakness of the strong resistance phenotype of SR_SA_, it is not possible to determine whether the resistance phenotype of the F_1_ is due to non-complementation at the *rph2* locus or an additive effect of the incompletely recessive allele previously attributed to the second locus [20].

A small proportion of the F_2_ progeny (15%–40%) are more sensitive than each of the parental strains as well as the F_1_ progeny. This is similar to the observation of a minor effect resistance allele in the previous SR_QLD_×SR_NSW_ cross. The F_2_ response curve demonstrates no clear indication of a plateau at ∼75% mortality. As *rph1* and *rph2* genes are responsible for the strong resistance of SR_QLD_, the lack of a plateau may either indicate that an *rph2* resistance allele is not present in SR_SA_ or that interpretation of the resistance phenotype is clouded by incomplete recessivity of *rph2* together with the phenotype of the minor effect gene. The data indicate that strong resistance of SR_SA_ is controlled by *rph1* plus a second major effect gene that may simply be a weak allele of *rph2*. While the data are consistent with this explanation, they do not rule out alternative explanations for the second major effect gene.

### Genetic complementation analysis of *SR_QLD_×SR2_QLD_*

**Figure 5:**
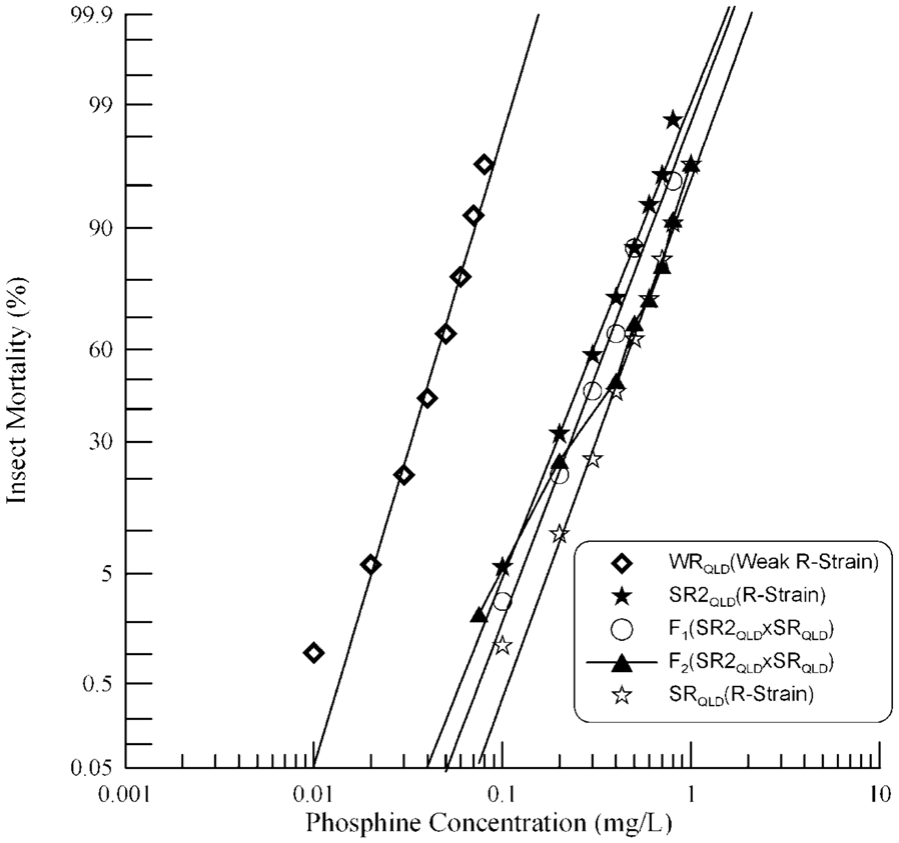
Resistance response of F_1_ hybrids and F_2_ progeny of a cross between two strongly resistant *R. dominica* strains from Queensland. Results are presented as log-dose mortality of the F_1_ hybrids and the F_2_ progeny with reference curves of the parental strains, SR2_QLD_ (R-Strain 2) and SR_QLD_ (R-Strain 1), and the weakly resistant strain from Queensland (WR_QLD_). Phosphine exposure was for 48 hours at 25°C and 70% r.h.

As with SR_NSW_ and SR_SA_, we also crossed SR_QLD_ with SR2_QLD_, a second strongly resistant strain collected from Queensland. Probit analysis revealed a linear response curve to phosphine exposure for each parental strain as well as for the F_1_ progeny (Figure 5), suggesting genetic homogeneity of each strain as well as the hybrid progeny. The resistance of the two parental strains differs by only 1.6 fold, with SR_QLD_ being more resistant.

The F_1_ progeny are highly resistant to phosphine exposure - slightly more than SR2_QLD_. This indicates that the second Queensland strain, SR2_QLD_, is not able to complement the original strongly resistant strain from Queensland, SR_QLD_, at either *rph1* or *rph2*. Thus, *rph1* and *rph2* are responsible for the strong resistance phenotypes of each strain. As with the preceding analyses, a small fraction of the progeny is unusually sensitive to phosphine exposure. This would appear to result from the lack of a minor dominant resistance factor contributed by one of the two parental strains. The fact that this minor, dominant factor has been apparent in crosses between SR_QLD_ and each of three independent strains, strongly implicates SR_QLD_ as the source of this additional resistance factor, a possibility that was first noted in Collins [3].

### Genetic complementation analysis of *SR2_QLD_×SR_NSW_*

**Figure 6:**
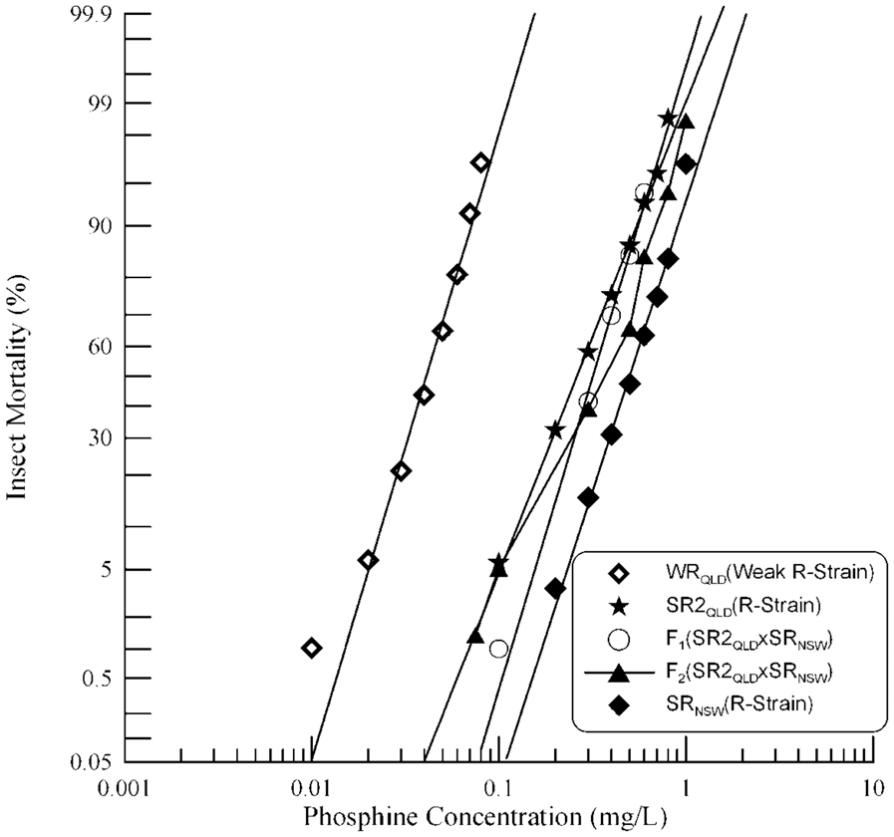
Resistance response of F_1_ hybrids and F_2_ progeny of a cross between strongly resistant *R. dominica* strains from Queensland and New South Wales. Results are presented as log-dose mortality of the F_1_ hybrids and the F_2_ progeny with reference curves of the parental strains, SR2_QLD_ and SR_NSW_, and the weakly resistant strain from Queensland (WR_QLD_). Phosphine exposure was for 48 hours at 25°C and 70% r.h.

A cross between SR2_QLD_ and SR_NSW_ was also made to determine allelic relationships between phosphine resistance genes in the two strains. Probit analysis revealed that the parental strains and the F_1_ progeny all show a homogeneous response to phosphine, indicated by linear phosphine resistance response curves (Figure 6). As with the cross with the F_1_ progeny of SR2_QLD_×SR_QLD_, the F_1_ progeny of SR2_QLD_×SR_NSW_ have a phenotype that is intermediate between the two strains. This indicates that resistance alleles of *rph1* and *rph2* are present in both strains.

The F_2_ progeny are mostly more resistant than the parental strain SR2_QLD_, though none is as resistant as SR_NSW_ (Figure 6). The F_1_ and F_2_ data together with results from crosses with the reference strain, SR_QLD_ suggest that the strong resistance phenotypes of SR2_QLD_ and SR_NSW_ are due to a resistance alleles of the *rph1* and *rph2* gene with the possibility of additional genes of minor effect.

### Genetic complementation analysis of *SR_SA_×SR2_QLD_*

**Figure 7:**
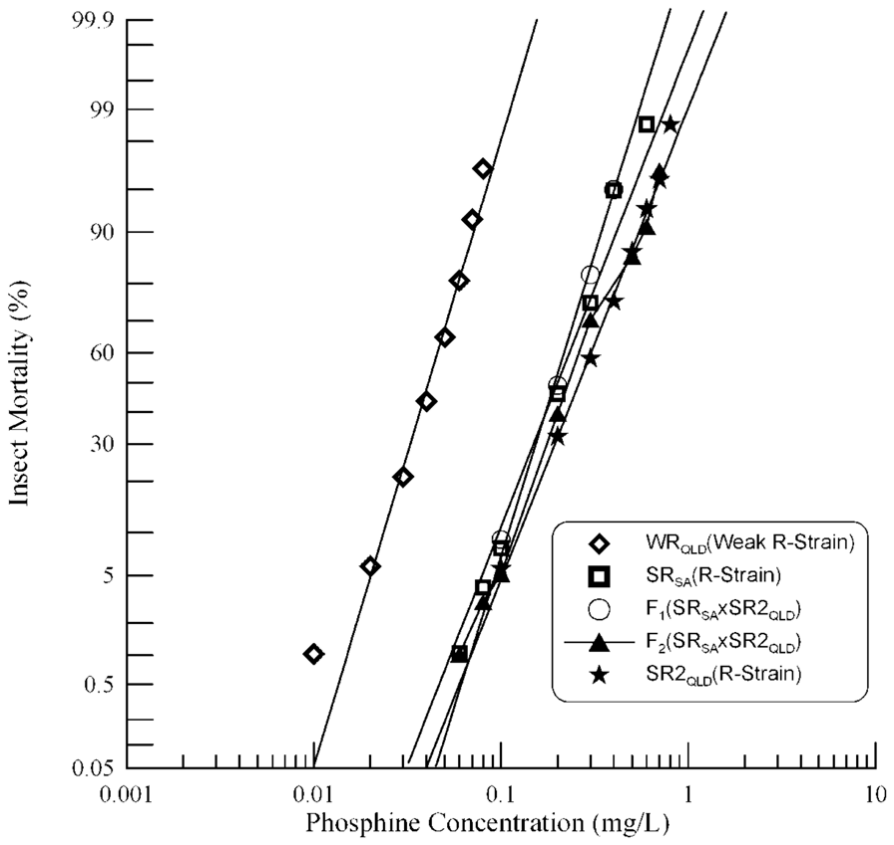
Resistance response of F_1_ hybrids and F_2_ progeny of a cross between strongly resistant *R. dominica* strains from Queensland and South Australia. Results are presented as log-dose mortality of the F_1_ hybrids and the F_2_ progeny with reference curves of the parental strains, SR2_QLD_ and SR_SA_, and the weakly resistant strain from Queensland (WR_QLD_). Phosphine exposure was for 48 hours at 25°C and 70% r.h.

We also crossed the second resistant strain from Queensland, SR2_QLD_ with the strain from South Australia, SR_SA_. Each of the parental strains and the F_1_ hybrid progeny showed linear response curves (Figure 7, Table 1). The resistance level of strain SR2_QLD_ is only 1.26 fold higher than strain SR_SA_, demonstrated by the very close proximity of their response curves. The resistance phenotype of the F_1_ progeny of this cross was similar to that of the parental strain SR_SA_. The F_2_ progeny were intermediate between the two parental strains (Figure 7). This indicates that alleles at the same loci, *rph1* and probably *rph2*, contribute to resistance in both SR_SA_ and SR2_QLD_ strains.

### Complex genetic crosses

The most reasonable explanation of the preceding genetic results is that all four highly resistant strains simply carry alternative alleles of the same two resistance genes. As demonstrated with the pairwise crosses, hybrids between such strains in the field should not lead to a dramatic increase in resistance to phosphine. The crosses that follow were designed to test the effect of combining all four resistance genotypes in a single strain and subjecting it to strong selection. The goal was to determine the resistance phenotype that would result from genetic combinations that had not been tested in the pairwise crosses. This includes testing the effects of resistance factors other than *rph1* and *rph2* after repeated selection for homozygosity of recessive alleles. The first of two strategies that were used consisted of setting up two pairwise crosses between strains that differed most strongly in their resistance phenotypes. The F_1_ progeny were then pooled to establish a “combined cross” strain. The second strategy consisted of setting up two pairwise crosses between strains that were most similar in their resistance phenotypes. This was followed by setting up a cross between hybrid F_1_s from the initial crosses to establish a “double hybrid” strain.

### Combined crosses (CC)

The mortality response data of each of the four parental strains used to establish the line with the combined genotype were subjected to probit analysis as presented in Table 1. The LC_50_ values of the parental strains are non-overlapping according to their 95% fiducial limits, indicating that the response phenotypes were unique. The strains that were initially crossed exhibited an approximately 2-fold difference in resistance levels (Table 1).

The combined strain was produced in two steps. Initially two single crosses were produced, SR_SA_×SR_QLD_ and SR2_QLD_×SR_NSW_. The progeny of these two crosses were then combined to establish an F_2_ generation. Selection for resistance was carried out at 0.5 mg/L phosphine in the F_3_ generation, 1.0 mg/L in the F_5_ generation and 1.0 mg/L in the F_7_ generation. Phosphine resistance of the combined cross was tested at the F_7_ and F_9_ generations, after having been selected for phosphine resistance two and three times, respectively. The F_7_ generation had a level of resistance 1.4 fold that of SR_NSW_ and in the F_9_ generation resistance had increased to 1.9 fold. Taken together, after 9 cycles of breeding and three selections with phosphine, the progeny of the combined crosses showed an increase in resistance less than two fold higher than that of the most strongly resistant parental strain SR_NSW_. This level of resistance is no more that previously observed for the minor effect gene contributed by SR_QLD_.

**Figure 8:**
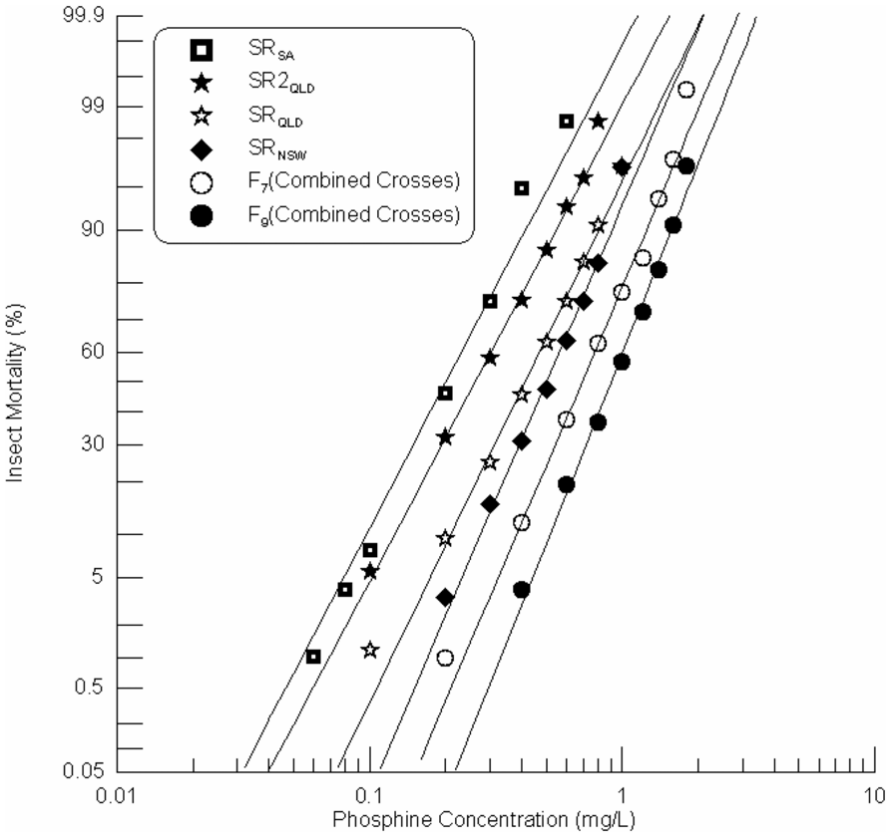
Resistance response of selected F_7_ and F_9_ progenies of a pooled hybrid (combined crosses) progeny of two single crosses between the strong resistant *R. dominica* strains, SR_QLD_×SR_SA_ and SR_NSW_×SR2_QLD_. Results are presented as log-dose mortality of the F_7_ and the F_9_ progenies with reference curves of the parental strains, SR_QLD_, SR2_QLD_, SR_NSW_ and SRSA. Phosphine exposure was for 48 hours at 25°C and 70% r.h.

Each of the response curves of the F_7_ and F_9_ progeny was linear suggesting that the line was quickly driven to genetic homogeneity by as few as two rounds of selection (Figure 8). The slopes of the response curves of the three weakest parental strains were nearly parallel (Figure 8), whereas the slope of SR_NSW_ more closely matched those of the F_7_ and F_9_ progenies of the combined cross (Figure 8). This suggests that a genetic factor from SR_NSW_ has been selected in the progeny.

### Double Crosses (DC)

We also combined all four strong resistance genotypes using an alternative strategy in which the two most strongly resistant strains, SR_NSW_ and SR_QLD_, were crossed as were SR_SA_ and SR2_QLD_. A double hybrid line was then produced by crossing individual offspring from each of the two single crosses. Selection for resistance was carried out in the F3, F5 and F7 generations as described for the combined crosses except that the single cross lines were also subjected to selection. The parental, single-cross and double-cross strains all exhibited homogeneous response curves with the exception of the single cross (SR_SA_×SR2_QLD_) at the F_9_ generation which gave a significant χ^2^ result (P<0.05) with a heterogeneity factor of 2.39 (Table 2).

**Table 2:** Probit analysis results of response of F_7_ and F_9_ progenies of single and double crosses of four *R. dominica* strong resistant strains to phosphine exposure.

*Strain (Cross)*	*n*	*Slope ± SE*	*LC_50_ (95% FL) (mg/L)*	*LC_99.9_ (mg/L)*	*df*	*χ^2^*	*P*
SR_QLD_	1353	4.49*±*0.35	0.415 (0.372–0.455)	2.026	6	9.805	0.133
SR_NSW_	1390	5.32*±*0.29	0.536 (0.504–0.565)	2.040	6	6.098	0.412
SR_SA_	2054	4.07*±*0.16	0.203 (0.193–0.214)	1.130	7	4.922	0.670
SR2_QLD_	1602	4.37*±*0.18	0.275 (0.259–0.290)	1.397	6	9.816	0.133
F_7_ (SR_QLD_×SR_NSW_)	2418	6.52*±*0.44	0.684 (0.646–0.719)	2.037	6	12.55	0.051
F_9_ (SR_QLD_×SR_NSW_)	2134	6.88*±*0.48	0.810 (0.770–0.847)	2.279	5	5.202	0.392
F_7_ (SR_SA_×SR2_QLD_)	2079	5.12*±*0.36	0.514 (0.473–0.551)	2.063	6	10.277	0.113
F_9_ (SR_SA_×SR2_QLD_)	2079	5.34*±*0.44	0.577 (0.527–0.622)	2.182	6	14.345	0.026*
F_7_ (DC)+	2178	5.64*±*0.27	0.785 (0.750–0.819)	2.773	8	9.224	0.324
F_9_ (DC)	2340	5.55*±*0.19	1.024 (0.997–1.049)	3.685	8	5.133	0.743

Estimated lethal concentrations, slopes and goodness-of-fit tests of probit lines of the parental strains, F_7_ and F_9_ progenies were presented. Insects were exposed to phosphine for 48 hours at 25°C and 70% r.h.

*Significant (P<0.05); **significant (P<0.01); ***significant (P<0.001).

+ DC = Double Crosses [F_1_ (SR_QLD_×SR_NSW_)×F_1_ (SR_SA_×SR2_QLD_)].

**Figure 9:**
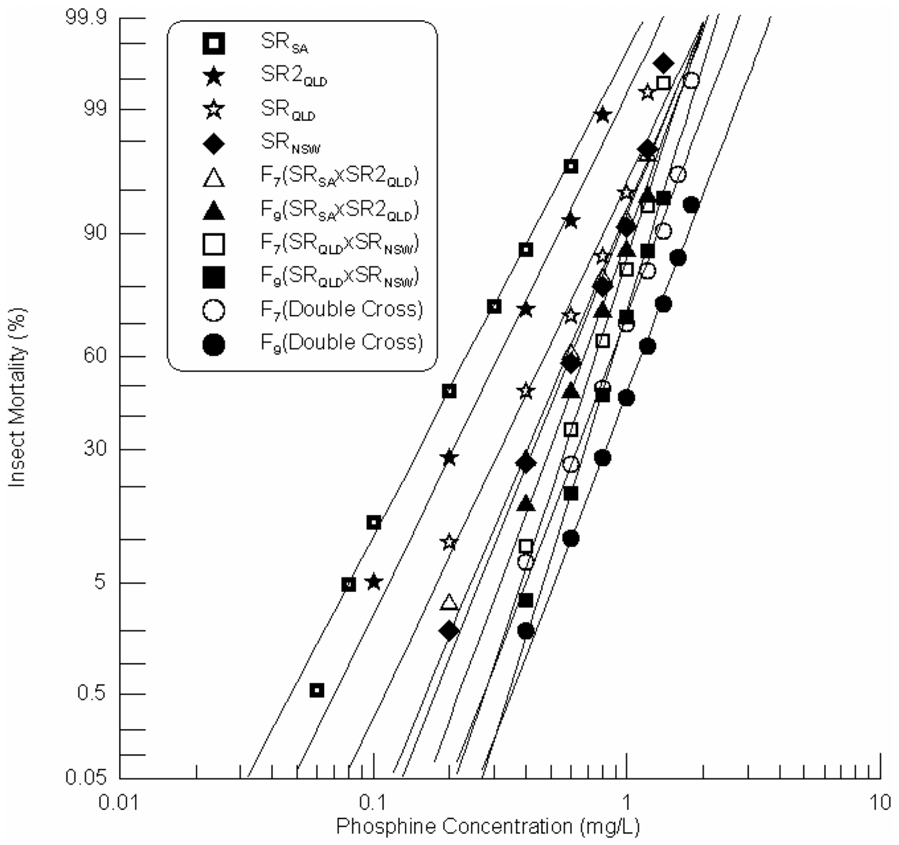
Resistance response of selected F_7_ and F_9_ progenies of double crosses between the strong resistant *R. dominica* strains from Australia. Results are presented as log-dose mortality of the F_7_ and the F_9_ progenies with reference curves of the F_7_ and F_9_ progenies the single crosses, SR_QLD_×SR_NSW_ and SR_SA_×SR2_QLD_, and the parental strains, SR_QLD_, SR2_QLD_, SR_NSW_ and SRSA. Phosphine exposure was for 48 hours at 25°C and 70% r.h.

All progenies of the single crosses exhibited a significantly higher level of resistance than the parental strains from which they were derived. Similarly, the F_9_ generation of the double cross was significantly more resistant than any of the parental lines, including the single cross lines from which the double cross lines were derived. Even the resistance level of the F_7_ progeny of the least resistant of the two single crosses SR_SA_×SR2_QLD_ is essentially equivalent to that of the strongest resistant strain SR_NSW_ given the overlapping LC_50_ (95% Fiducial Limit) (Table 2, Figure 9). These results are tabulated as resistance factors (ratios of LC_50_ values) in Table 3.

**Table 3:** Resistance factor of phosphine selected F_7_ and F_9_ progenies of both single and double crosses relative to their respective parental strains/lines.

Strain/Cross*	Single/Double Crosses*					
	F_7_-SC1	F_9_-SC1	F_7_-SC2	F_9_-SC2	F_7_-DC	F_9_-DC
SR_QLD_	1.65	1.95			1.89	2.47
SR_NSW_	1.28	**1.51**			1.46	**1.91**
SR_SA_			2.53	2.84	3.87	5.04
SR2_QLD_			1.87	**2.10**	2.85	3.72
F_7_-SC1					1.15	1.50
F_9_-SC1					0.97	1.26
F_7_-SC2					1.53	1.99
F_9_-SC2					1.36	1.77

* SC1 = Single Cross 1 (SR_QLD_×SR_NSW_), SC2 = Single Cross 2 (SR_SA_×SR2_QLD_), DC = Double Crosses (F_1_-SC1×F_1_-SC2). The resistance factor in each cell is calculated as the LC_50_ of the strain indicated in the column header divided by the LC_50_ of the strain indicated in the first column.

The F_9_ (selected 3 times) progeny of each single and double cross had a resistance factor from 1.5 to 2.1 times that of the most resistant parental strain from which it was derived (shown in bold in Table 3). The resistance factor of the double cross strain increased between the F_7_ and F_9_ generations by 30%. Interestingly, this was simply the sum of the increases of the two single cross strains (12% and 18%). The small changes and similarity between the strains indicates that the genetic interactions are simply additive as would be expected if sensitive alleles of minor effect genes were being progressively eliminated.

## Discussion

### Allelic relationships between phosphine resistance genes

Due to the growing threat of resistance across the world, understanding the genetics of phosphine resistance will provide globally significant insight into effective phosphine resistance management strategies. The present work is a continuation of previous research in which we found that the *rph1* gene contributed to the strong resistance phenotype in three of the strains that have been re-examined in this study [20]. We also demonstrate that an allele of *rph1* contributes to resistance in a fourth strongly resistant strain, SR2_QLD_. In addition to *rph1*, a second genetic factor that is semi-dominant contributes to the strong resistance phenotype of all four strains SR2_QLD_, SR_QLD_, SR_NSW_ and SR_SA_. This second gene was previously characterised in SR_QLD_ and is named *rph2*.

Crosses between the strongly resistant strains provided additional insight into relationships between phosphine resistance alleles from the four resistant strains. These results confirm that the *rph1* gene contributes to resistance in each of SR_NSW_ and SR_SA_ [20], SR_QLD_ [18], [19] and SR2_QLD_. The results also suggest that the incompletely recessive synergistic factor first noted in SR_QLD_ [3] is a synergistic resistance factor in each of the four strains. Schlipalius et al. [19] proposed that the evolution of resistance was constrained by the fact that the *rph2* gene is relatively insignificant as a resistance locus in the absence of the resistance allele at *rph1*. We now provide evidence that the evolution of resistance in independent outbreaks is constrained to just two major genes in *R. dominica*. In addition, a minor effect, dominant resistance factor was identified that contributes about 2-fold resistance. It is interesting to note that strong resistance toward phosphine in *Tribolium castaneum* is also due to two synergistically interacting genetic factors, suggesting that elements of resistance may be shared between species as well [25].

### Combining resistance genotypes to establish maximal resistance levels

We also combined resistance genotypes from all four strongly resistant strains to determine if enhanced resistance to phosphine could be produced. We employed both mass-combined and defined double-hybrid crossing strategies to combine all resistance alleles in a single population. The progenies of both mass-combined and double-hybrid crosses exhibited increased levels of resistance relative to the parental strains after either two or three rounds of selection for phosphine resistance. The highest resistance levels obtained from the selected progenies of both mass-combined and double-hybrid crosses were 1.8 and 1.9-fold higher than the most resistant parental strain, SR_NSW_. This level of increase in resistance can most likely be attributed to genes of minor effect contributed by the genetic backgrounds of the parental strains.

The present study confirms and extends our previous understanding that the *rph1* gene is a common contributor to resistance. We also present strong evidence that *rph2* together with *rph1* explains nearly all of the strong resistance phenotype. A few additional resistance factors appear to contribute to the resistance of the strains that we have investigated in this paper. The effect of such minor genes is to increase resistance about 2-fold beyond the level of the most strongly resistant parental strain. The overarching hypothesis from this work is that limited genetic mechanisms are responsible for all strong resistance outbreaks in *R. dominica* and possibly other species as well. These results, however, do not rule out the possibility that novel resistance genes may eventually be isolated from *R. dominica* or other species in Australia or elsewhere. In this regard, the extremely high level of phosphine resistance recently observed in *Cryptolestes ferrugineus* is a prime candidate for further study [26].

We have now demonstrated that four strongly phosphine resistant strains of *R. dominica* each carry alleles of *rph1* and *rph2* that are responsible for nearly the entire resistance phenotype. This finding is quite remarkable given that phosphine is a very small and reactive molecule that could potentially have many target sites within a cell. Nevertheless, our findings suggest that the job of monitoring and managing resistance will be much more manageable than could have been the case if the genetic basis of resistance was more complex. It remains to be determined whether the same genetic basis of resistance extends to other species. If this is the case, it will allow development of a universal marker that will be useful in efficient monitoring and management of phosphine resistance.

The fact that homozygosity of *rph1* alone confers only weak resistance to each of the four strains, suggests that as with SR_QLD_, the strong resistance phenotype is due to a synergistic interaction between *rph1* and *rph2*. This means that the phosphine resistance problem can be alleviated by strategies that disrupt resistance caused by either *rph1* or *rph2*, as such strategies need not target both mechanisms to be effective.

Development of diagnostic markers for monitoring resistance will be greatly facilitated by cloning of the resistance gene. Cloning of the gene will also allow comparative genetic analysis of resistance between species. Identification of the gene will also facilitate detailed genetic studies into the mode of action and mechanisms of resistance toward phosphine. This type of work is made more valuable by the outcome of the current study, which suggests that the resistance genes that we have identified may define the extent of the problem that will be faced by the grains industry.
